# Effects of Dietary Supplementation with Eucalyptus Essential Oil and Soy Isoflavones on the Growth Performance, Intestinal Health and Meat Quality of Weaned Meat Rabbits

**DOI:** 10.3390/ani15192890

**Published:** 2025-10-02

**Authors:** Chaowu Fu, Rao Li, Zhengpu Wei, Yurong Yang, Yan Zhang, Yibao Jiang

**Affiliations:** 1 College of Animal Science and Technology, Henan Agricultural University, Zhengzhou 450046, China; chaowufu@stu.henau.edu.cn (C.F.);; 2 College of Veterinary Medicine, Henan Agricultural University, Zhengzhou 450046, China

**Keywords:** rabbit, eucalyptus essential oil, soy isoflavones, growth performance, intestinal health, meat quality

## Abstract

This study investigated the effects of individual and combined supplementation of eucalyptus essential oil (EEO) and soy isoflavones (SI) on the growth performance, intestinal health and meat quality of weaned meat rabbits. A total of 120 Ira female rabbits were stratified by body weight (BW) and randomly allocated into four treatment groups. The rabbits were fed for 35 days using (a) basal diet (Control), (b) basal diet + 150 mg/kg EEO, (c) basal diet + 25 mg/kg SI, or (d) basal diet + 150 mg/kg EEO + 25 mg/kg SI. The results showed that supplementing EEO and SI in the diet had a synergistic effect on production performance by increasing BW and average daily gain and reducing the feed intake/gain ratio. The EEO + SI group exhibited the most pronounced improvement in growth performance. Compared with the Control group, individual or combined supplementation significantly increased both semi-clean slaughter and total eviscerated slaughter rates, and showed a synergistic effect when supplemented together. The antioxidant capacity of the rabbits was enhanced, with the EEO + SI group showing the largest improvement. Serum biochemical analysis revealed that the combined treatment improved hepatic and cardiovascular functions. Additionally, we observed that the combined addition of EEO and SI has a synergistic effect on increasing the height of intestinal villi and the villus height/crypt depth ratio. Moreover, EEO and SI synergistically improved meat quality by elevating pH value, water-holding capacity, tenderness, and fat proportion. Notably, supplementation of EEO and SI in the diet regulated the composition of the intestinal microbiota, demonstrating increased relative abundance of beneficial *Bacteroides* and *Lachnospiraceae_NK4A136_group*. In conclusion, the combination of EEO and SI outperformed individual supplements in promoting growth and intestinal barrier function, potentially through enhancing antioxidant activity, attenuating oxidative stress, protecting the hepatic/cardiovascular systems, and improving intestinal morphology and microbial composition, thereby collectively enhancing growth performance, slaughter traits, and meat quality.

## Simple Summary

The weaning period is a highly sensitive stage in meat rabbit production, where sudden dietary changes and environmental shifts may trigger gastrointestinal disorders, thereby impairing production performance. This study evaluated the effects of dietary supplementation with eucalyptus essential oil and soybean isoflavones in weaned meat rabbits. The results demonstrated that individual or combined supplementation of eucalyptus essential oil and soybean isoflavones improved growth performance, intestinal health and meat quality; enhanced slaughter yield and meat quality; and augmented antioxidant capacity. The most pronounced effects were observed with combined supplementation.

## 1. Introduction

Weaning is a critical phase in meat rabbit production, as it is frequently accompanied by gastrointestinal disorders, diarrhea, microbial dysbiosis, and compromised intestinal barrier integrity that collectively impair growth performance [1]. High stress levels during this stage may lead to malabsorption and immunosuppression, ultimately affecting overall production efficiency and the animals’ welfare. To reduce the dependency on antibiotics, natural feed additives such as plant essential oils and phytoextracts are promising alternatives. Among these, eucalyptus essential oil (EEO) and soy isoflavones (SI) have demonstrated superior potential in enhancing weaned animal growth and gut health due to their antimicrobial, anti-inflammatory, and antioxidant properties.

EEO, primarily composed of 1,8-cineole, has broad-spectrum antimicrobial, anti-inflammatory, and antioxidant properties [2]. Studies have demonstrated that dietary supplementation of rabbits with eucalyptus leaves significantly reduces pathogenic bacterial loads (e.g., *Escherichia coli* and *Salmonella*) in the small intestine while improving the intestinal morphology, thereby enhancing nutrient digestibility and growth performance [3,4]. Additionally, rabbits supplemented with eucalyptus leaf extract exhibited reduced cecal pathogenic bacteria and improved meat quality [4]. Studies have confirmed that supplementation of dietary oregano essential oil in rabbits upregulated the expression of intestinal tight junction proteins, strengthened gut barrier function, and reduced the incidence of diarrhea [5]. Essential oils also increased average daily gain and apparent nutrient digestibility in weaned piglets while reducing *E. coli* colonization in the rectum by 42.7%, a mechanism mediated by improved villus height/crypt depth (V/C) ratio and elevated levels of serum immunoglobulins [6]. SI, functioning as phytoestrogens, possess multifaceted bioactive properties, including antimicrobial, antiviral, anti-inflammatory, antioxidant, and antitumor activities [7,8]. Studies have shown that supplementing SI to female rabbits during lactation can significantly improve the growth performance and serum immune indicators of their offspring, as well as enhance the morphology of the small intestine. These effects are related to the antioxidant and anti-inflammatory properties of SI [9]. Furthermore, SI downregulated intestinal NF-κB signaling pathways, alleviated oxidative stress markers, and preserved barrier integrity through reinforcement of claudin-5 [9]. These findings collectively indicate that strategic supplementation of EEO and SI may enhance the growth performance and health status of rabbits.

Although EEO or SI have demonstrated their efficacy in individual applications, the synergistic interaction between EEO and SI remains unexplored territory. Current research predominantly focuses on singular supplements or porcine models [10,11]. Therefore, a systematic evaluation of EEO and SI in rabbit production is needed for developing high-efficiency natural supplements. This study aimed to comprehensively evaluate the effects of EEO and SI supplementation on the growth performance, meat quality intestinal health of weaned meat rabbits to provide a foundation for developing natural additive strategies, ultimately facilitating antibiotic-free production and enhancing sustainable rabbit farming.

## 2. Materials and Methods

### 2.1. Ethical Statement

This study strictly adhered to China’s Regulations on the Management of Laboratory Animals and the internationally recognized 3R principles (Replacement, Reduction, and Refinement). All experimental protocols (Approval No. HNND2023031125) were reviewed and approved by the Animal Ethics Committee of Henan Agricultural University.

### 2.2. Experimental Material

EEO and SI were provided by Longchang Animal Health Products Co., Ltd., Dezhou, China.

### 2.3. Animal Breeding and Experimental Design

To eliminate the potential confounding effect of sex, Ira female rabbits (French genetic origin, reared in Nanyang, Henan, China) were used exclusively. All rabbits were individually housed in stainless steel cages under Controlled environmental conditions. The ambient temperature was maintained at 15–20 °C, with a relative humidity of 55–65%, consistent with standard husbandry protocols. Rabbits were fed twice daily (08:00 and 20:00), and they had ad libitum access to water and feed. The rabbits (45 days old, average body weight = 1.16 ± 0.05 kg) were randomly divided into four treatment groups, with five replicates in each group and six rabbits in each replicate (*n* = 30/group). The treatment groups were as follows: (a) Control: basal diet formulated per NRC (1977) rabbit nutrient requirements (Table 1), (b) EEO: basal diet + 150 mg/kg EEO, (c) SI: basal diet + 25 mg/kg SI, and (d) EEO + SI: basal diet + 150 mg/kg EEO + 25 mg/kg SI. EEO and SI were used as supplements in this experiment, without altering the diet formula. The supplementation dose of EEO was determined based on the effective immunomodulatory dose established in rabbit studies and the maximum safe level of EEO documented in rabbits [2,12]. The supplementation dose of SI was derived from effective doses reported in rabbit models and doses that improved growth performance and digestive function in poultry studies [13,14]. In light of the above and following the supplier’s recommendations, both supplements were administered at the lower end of the effective dose range to mitigate potential risks associated with higher dosages.

### 2.4. Determination of Growth Performance

Before the start of the trial, 38-day-old rabbits were acclimated for a 7-day pre-experimental period. The entire experiment was divided into two stages, namely the early fattening period (45–65 days) and the late fattening period (66–80 days). Body weights (BWs) were recorded at the initiation of the formal trial (45 days of age), with subsequent measurements taken at 65 and 80 days of age. Throughout the experiment, the rabbits underwent an 8-h fasting period before each weighing session. Additionally, the actual feed intake for each replicate was recorded daily and used to calculate growth performance parameters, including average daily feed intake (ADFI), average daily gain (ADG), and feed intake/gain (F/G). The calculation formulas are as follows:

$$\mathrm{ADFI}\;(g)=\frac{\mathrm{Total}\;\mathrm{feed}\;\mathrm{consumption}\;\mathrm{per}\;\mathrm{replicate}\;}{\mathrm{Number}\;\mathrm{of}\;\mathrm{rabbit}\times \mathrm{Number}\;\mathrm{of}\;\mathrm{test}\;\mathrm{days}}$$

$$\mathrm{ADG}\;(g)=\frac{\mathrm{Final}\;\mathrm{body}\;\mathrm{weight}-\mathrm{Initial}\;\mathrm{body}\;\mathrm{weight}}{\mathrm{Test}\;\mathrm{days}}$$

$$F/G=\frac{\mathrm{ADFI}}{\mathrm{ADG}}$$

### 2.5. Sample Collection

On day 80, three rabbits per group with comparable BWs were selected for sampling. Blood samples were collected via auricular venipuncture using anticoagulant tubes, and serum was subsequently isolated by centrifugation (3500× *g*, 15 min, 4 °C) for determination of serum biochemical parameters and antioxidant capacity. After humane euthanasia, the carcasses and visceral organs were weighed to assess slaughter performance according to WRSA standard criteria. The longissimus dorsi muscle from the left side of each carcass was excised for analysis of meat quality traits and nutrient composition. Mid-segments of the duodenum, jejunum, and ileum were immediately fixed in 4% paraformaldehyde for morphological examination. Finally, cecal digesta were aseptically collected into cryovials, flash-frozen in liquid nitrogen, and stored at −80 °C for 16S rDNA sequencing targeting the V3–V4 hypervariable regions.

### 2.6. Determination of Slaughter Performance

Three healthy rabbits per group were randomly selected after a 12-h fasting period for BW measurement and humane slaughter. Post-slaughter measurements included the half-eviscerated weight, the full-eviscerated weight, the semi-clean slaughter rate, and the total eviscerated slaughter rate [15].

### 2.7. Determination of Muscle Quality and Nutrient Composition

Upon conclusion of the feeding trial, physicochemical analysis of the muscle was conducted according to standard methods. The measured parameters included pH value, expressible loss, drip loss, cooking loss, shear force value, and meat composition [16].

### 2.8. Determination of Serum Biochemical Indicators and Serum Antioxidant Capacity

Serum biochemical parameters were quantified using a BK-280 fully automated biochemistry analyzer (BIOBASE Co., Ltd.; Jinan, China). Serum antioxidant capacity was determined using a commercially available kit (Nanjing Jiancheng Bioengineering Institute; Nanjing, China) according to the manufacturer’s protocols.

### 2.9. Morphological Analysis of the Small Intestine

Intestinal segments (duodenum, jejunum, and ileum) from three rabbits per group were fixed in 4% paraformaldehyde. Following dehydration and paraffin embedding, 4-μm-thick tissue sections were prepared and stained with hematoxylin and eosin (H&E). Six fields of view per intestinal section were systematically selected for morphometric analysis. Villus height and crypt depth were measured using a calibrated ocular micrometer, with mean values derived from all measurements. The V/C ratio was subsequently calculated. Morphometric assessments were performed using a LEICA DM2000 microscopy system (Leica Microsystems, Wetzlar, Germany) equipped with image analysis software (Leica Application Suite V4.12). The calculation method for the V/C ratio is as follows:

$$V/C\;\mathrm{ratio}=\frac{Villus\;height}{Crypt\;depth}$$

### 2.10. Analysis of the Cecal Microbiota

Cecal digesta were aseptically collected for microbial total DNA extraction using SDS-based lysis buffer coupled with proteinase K digestion. The hypervariable V3–V4 region of the 16S rRNA gene was amplified using the fusion primers 338F (5′-ACTCCTACGGGAGGCAGCAG-3′) and 806R (5′-GGACTACHVGGGTATCTA AT-3′) PCR amplicons underwent size selection and purification via the AMPure XP magnetic bead system (Thermo Fisher Scientific, Waltham, MA, USA). Sequencing libraries were prepared and subjected to high-throughput sequencing on an Illumina platform (Illumina, San Diego, CA, USA). The raw sequencing data underwent preliminary quality screening based on Phred scores (Q ≥ 30), with problematic samples being re-sequenced. Primer sequences were trimmed using Cutadapt (v3.5), and reads with mismatched primers were discarded. Subsequent processing employed the DADA2 pipeline (v1.22.0) in QIIME (2022.2 release) for quality control, denoising, paired-end merging, and chimera removal [17]. Rarefaction curves were generated using the coverage index derived from alpha diversity analysis to assess whether sequencing depth sufficiently captured the full taxonomic diversity of samples and to validate the rationality of sequencing data. Alpha diversity metrics, including the Shannon index, Simpson index, Chao1 richness estimator, Ace richness estimator, and observed richness (Sob), were calculated from the OTU abundance table to quantify microbial community diversity and species richness. Furthermore, beta diversity was estimated by calculating Bray–Curtis dissimilarity distances between the experimental groups and controls.

### 2.11. Statistical Analysis

In this study, data normality and homogeneity of variance were assessed using the Shapiro–Wilk and Levene tests (SPSS 26.0). One-way ANOVA with Duncan’s post hoc test was applied for normally distributed data; the Kruskal–Wallis test was employed for non-normally distributed data. Results are expressed as mean ± standard deviation. Statistical significance was defined as *p* < 0.05.

Data processing employed the Bliss independence model for quantification of additive/synergistic effects to determine whether the combined application of EEO and SI resulted in additive or synergistic interactions. The calculation method is as follows:

$$Q=\frac{E_{AB}}{\left(E_{A}+E_{B}-E_{A}\times E_{B}\right)}$$

Note: *E_A_*, *E_B_*, and *E_AB_* represent the relative changes (increases or decreases) in the EEO group, SI group, and EEO + SI group, respectively, compared to the control group. *Q* > 1.15 represents a synergistic effect; 0.85 ≤ *Q* ≤ 1.15 represents the additive effect; *Q* < 0.85 indicates an antagonistic effect. If the *Q* value is negative, it has no biological significance and no statistical analysis will be conducted [18].

## 3. Results

### 3.1. Growth Performance

The effects of different treatments on growth performance are presented in Table 2. Compared with the control group, the EEO + SI group exhibited a significant increase in body weight at 65 days (*p* < 0.05). At 80 days, all treatment groups showed significantly higher BW compared to the control (*p* < 0.05). Furthermore, the BW of the EEO + SI group was significantly greater than that of the SI group at 80 days (*p* < 0.05). At 45–65 days, the ADG of all treatment groups was significantly higher than Control group (*p* < 0.05). Furthermore, the F/G of all treatment groups showed a significant reduction (*p* < 0.05). Additionally, in the period of 45–65 days, the ADG of the EEO + SI group was significantly higher than that of the EEO group, increasing by 9.22% (*p* < 0.05). At 45–80 days, ADG was significantly increased in all treatment groups relative to the Control group (*p* < 0.05), with ADG in the EEO + SI group being significantly higher than that in the SI group, and it increased by 6.05% compared to the SI group (*p* < 0.05). Additionally, during this phase, F/G in the EEO and EEO + SI groups was significantly lower than that in the Control group (*p* < 0.05). During the 66–80 days period, no significant differences were observed in ADG, ADFI, or F/G between any treatment groups and the Control group (*p* > 0.05). The supplementation of EEO and SI in feed exerted a synergistic effect on BW at 65 and 80 days (*Q* > 1.15). Moreover, synergistic effects were also observed on ADG and F/G during the periods of 45–65 days and 45–80 days (*Q* > 1.15).

### 3.2. Slaughter Performance

When EEO and SI were supplemented individually or in combination in the diet, all treatment groups exhibited significantly increased half-eviscerated weight, all eviscerated weight, semi-clean slaughter rate, and total eviscerated slaughter rate compared to the Control group (*p* < 0.05). Moreover, the combined supplementation demonstrated a synergistic effect on semi-clean slaughter rate and total eviscerated slaughter rate (*Q* > 1.15) (Table 3).

### 3.3. Muscle Quality and Nutrient Composition

The pH_45min_ in the EEO + SI group was significantly higher than that in the Control group (*p* < 0.05). Additionally, all treatment groups exhibited a significant decrease in drip loss (*p* < 0.05). Specifically, the EEO + SI group reduced drip loss by 24% and 18.36% compared to the EEO group and SI group, respectively. Furthermore, the shear force value in the EEO + SI group was significantly lower than that in the Control group (*p* < 0.05), and it was 9.46% and 5.42% lower than that of the EEO group and the SI group, respectively. Notably, all treatment groups showed a significant increase in ether extract (*p* < 0.05). Moreover, the combined supplementation of EEO and SI in the diet demonstrated a synergistic effect on pH_45min_, drip loss, shear force value, and ether extract (*Q* > 1.15) (Table 4).

### 3.4. Serum Biochemical Indices

The effects of dietary supplementation with EEO and SI, individually or in combination, on serum biochemical parameters are presented in Table 5. Compared with the Control group, the EEO + SI group exhibited significantly decreased levels of ALT, AST, and LDH (*p* < 0.05). Notably, all experimental groups showed significantly reduced TG concentrations relative to the Control group (*p* < 0.05). Among them, the AST level in the EEO + SI group was significantly lower than that in the EEO group and SI group (*p* < 0.05). In the EEO + SI group, compared to the EEO group and SI group, respectively, ALT levels decreased by 11.71% and 14.02%, AST levels by 27.59% and 16.52%, and LDH levels by 11.51% and 6.02%. Furthermore, the combined supplementation of EEO and SI in the diet of meat rabbits has a synergistic effect on ALT, AST, LDH and TG (*Q* > 1.15). These results indicate that the combined dietary supplementation of EEO and SI leads to superior improvement in serum biochemical parameters compared to individual supplementation.

### 3.5. Serum Antioxidant Capacity

All experimental groups exhibited significantly elevated T-AOC and reduced MDA levels compared with the Control group (Table 6; *p* < 0.05). The MDA level in the EEO + SI group was significantly lower than that in the EEO group (*p* < 0.05), with reductions of 13.03% and 12.32% compared to the EEO and SI groups, respectively. Compared with the Control group, the GSH-Px activity in the EEO + SI group was significantly increased (*p* < 0.05). Furthermore, the GSH-Px activity in the EEO + SI group showed a 7.13% and 7.76% elevation compared to the EEO group and SI group, respectively. In terms of serum antioxidant capacity, the combined supplementation of EEO and SI has a synergistic effect on T-AOC, MDA and GSH-Px (*Q* > 1.15). This indicates that the combined supplementation of EEO and SI has achieved the greatest improvement in serum antioxidant capacity.

### 3.6. Small Intestinal Morphology

**Figure 1:**
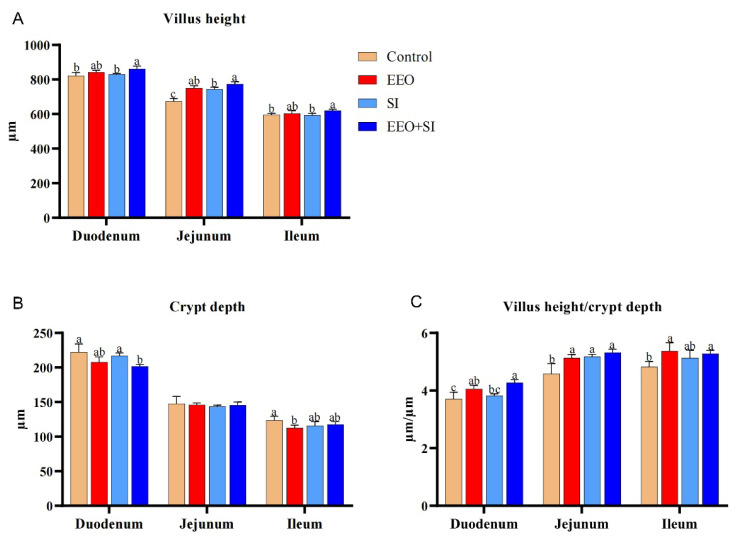
The effects of dietary supplementation with eucalyptus essential oil and soy isoflavones on the morphology of the small intestine of meat rabbits at the end of the experiment (*n* = 3 per group). (**A**) Villus height, *Q*_Duodenum_ = 1.93; *Q*_Jejunum_ = 1.42; *Q*_Ileum_ = 3.16. (**B**) Crypt depth, *Q*_Duodenum_ = 1.47; *Q*_Jejunum_ = 1.37; *Q*_Ileum_ = 0.60. (**C**) Villus height/crypt depth ratio, *Q*_Duodenum_ = 1.67; *Q*_Jejunum_ = 1.46; *Q*_Ileum_ = 0.89. Values are expressed as mean ± standard deviation (SD). Different letters indicate statistically significant differences (*p* < 0.05). *Q* > 1.15 represents a synergistic effect.

**Figure 2:**
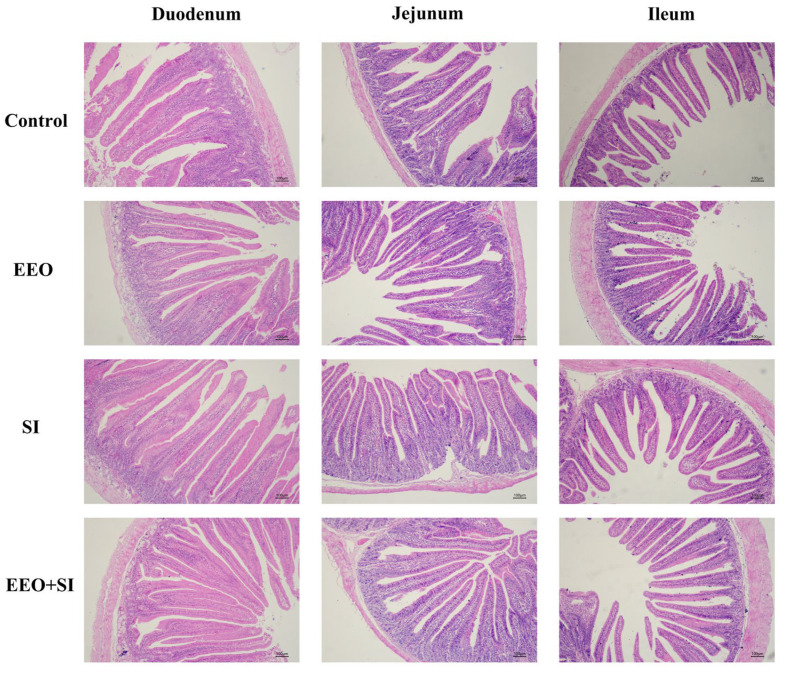
The effects of dietary supplementation with eucalyptus essential oil and soy isoflavones on the morphology of the small intestine of meat rabbits at the end of the experiment (100×). Representative hematoxylin and eosin (H&E)-stained sections of three intestinal segments (duodenum, jejunum, and ileum) from the Control, EEO, SI, and EEO + SI groups. Scale bar = 100 µm.

The effects of individual or combined dietary supplementation with EEO and SI on small intestinal morphology are illustrated in Figure 1 and Figure 2. In the duodenal segment, the villus height in the EEO + SI group was significantly higher than that in the Control group and the SI group (*p* < 0.05). Moreover, the crypt depth in the EEO + SI group was significantly lower than that in the Control group and SI group (*p* < 0.05). The V/C ratio in both the EEO group and EEO + SI group was significantly higher than that in the Control group (*p* < 0.05), and the V/C ratio in the EEO + SI group was significantly higher than that in the SI group (*p* < 0.05).

In the jejunal segment, the villus height and the V/C ratio in all experimental groups were significantly increased compared with those in the Control group (*p* < 0.05). Moreover, the villus height in the EEO + SI group was significantly higher than that in the SI group (*p* < 0.05).

In the ileum, the villus height was significantly higher in the EEO + SI group compared to the Control and SI groups (*p* < 0.05). The crypt depth in the EEO group was significantly lower than that in the Control group (*p* < 0.05). Additionally, the V/C ratio was significantly elevated in both the EEO and EEO + SI groups relative to the Control group (*p* < 0.05).

Dietary co-supplementation of EEO and SI demonstrated synergistic effects on villus height, crypt depth, and V/C ratio in the duodenum and jejunum, as well as on villus height in the ileum (*Q* > 1.15).

### 3.7. Microbial Composition of Cecum Contents

#### 3.7.1. Dilution Curves of the Intestinal Microbiota

**Figure 3:**
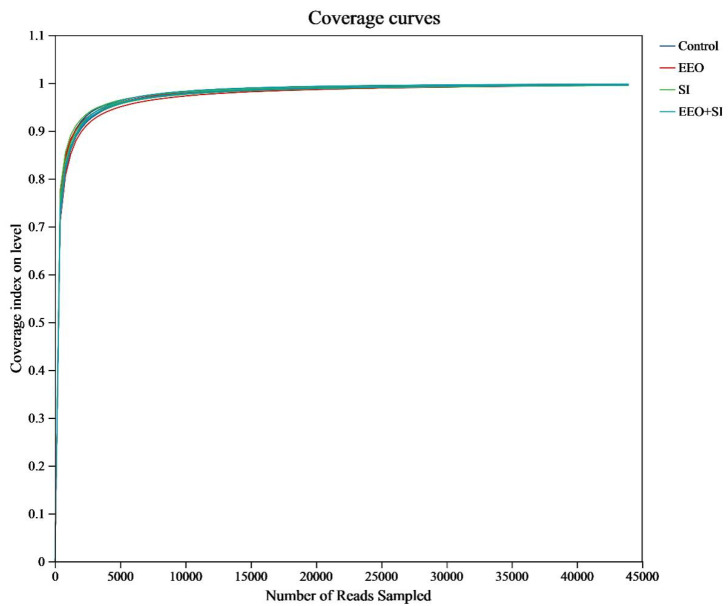
The coverage index as a function of the number of reads.

The coverage indices from the alpha diversity analysis were used to generate rarefaction curves that indicated the sequencing coverage efficiency across sample groups. The dilution curves for all groups plateaued asymptotically (Figure 3), indicating that the sequencing depth was sufficient to capture the vast majority of microbial taxa within each sample. This demonstrated the robustness and representativeness of the sequencing data.

#### 3.7.2. Alpha Diversity Analysis

**Figure 4:**
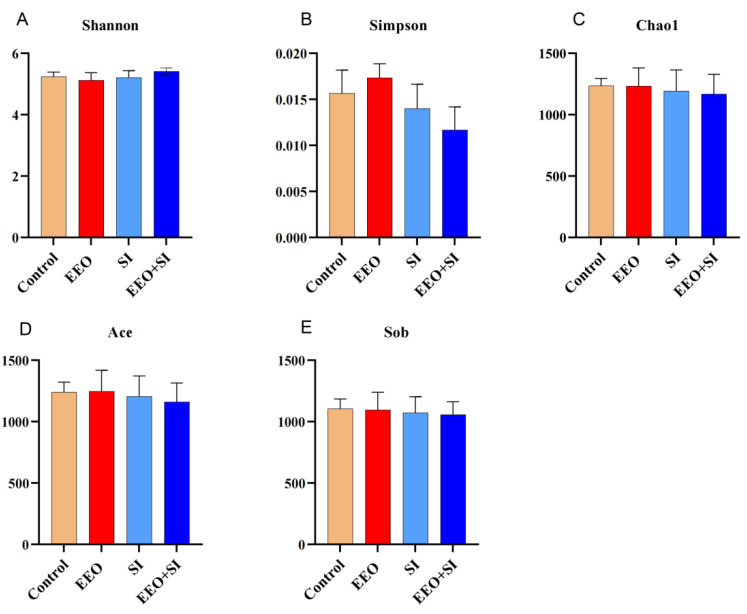
Alpha diversity analysis. (**A**–**E**) Shannon, Simpson, Chao1, Ace and Sob indices were used to assess alpha diversity.

The alpha diversity metrics indicated no significant differences among the treatment groups (Figure 4A–E; *p* > 0.05), demonstrating that dietary interference did not alter the alpha diversity.

#### 3.7.3. Beta Diversity Analysis of the Cecal Microbiota

**Figure 5:**
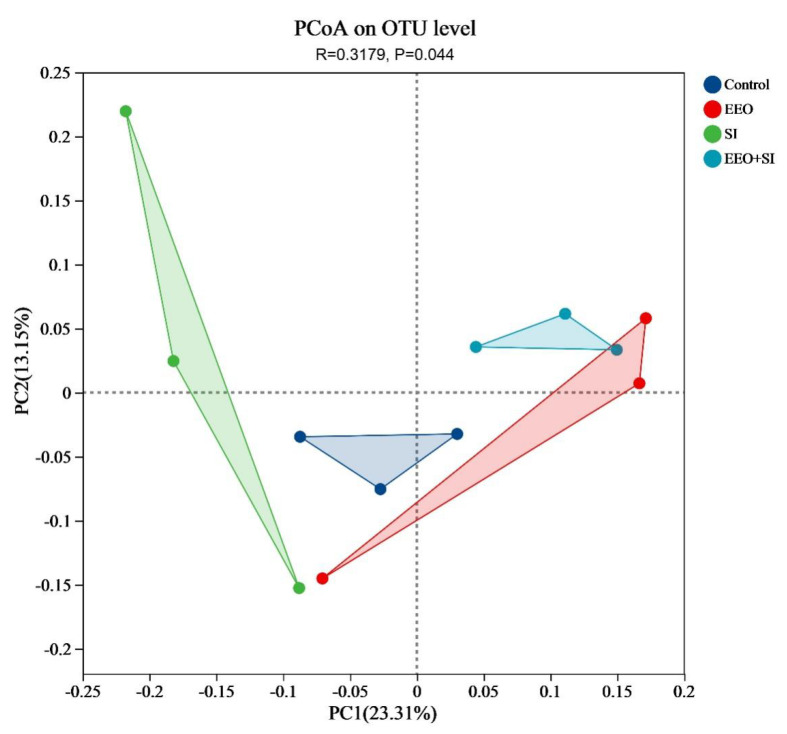
Principal coordinate analysis (PCoA) based on the unweighted UniFrac distance matrix, revealing significant separation between the experimental groups.

The two principal coordinates (PC1 and PC2) accounted for 23.31% and 13.15% of the total variance, respectively. Distinct clustering patterns were observed between all treatment groups and the Control group, indicating statistically significant differences in microbial community structure (Figure 5, R = 0.3179, *p* = 0.044).

#### 3.7.4. Microbiota in Cecum Contents

**Figure 6:**
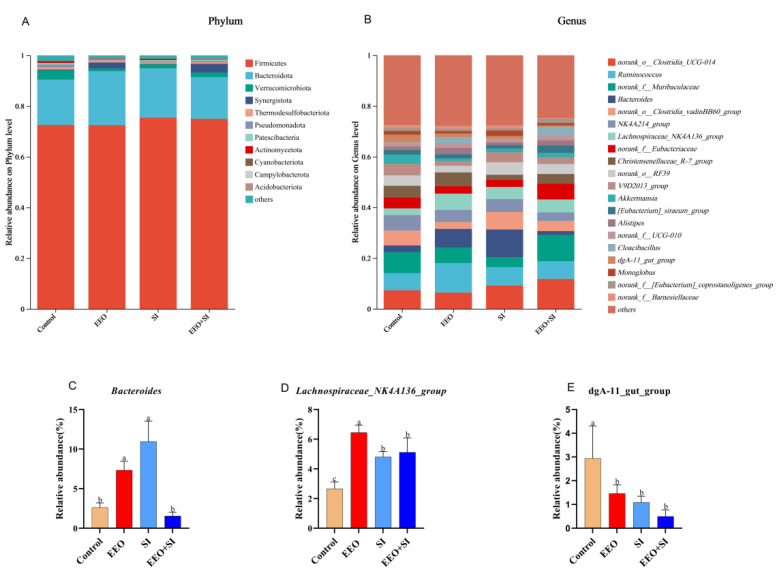
The effects of dietary supplementation with eucalyptus essential oil and soy isoflavones on the morphology of the microbiota of meat rabbits at the end of the experiment (*n* = 3 per group). The relative abundances of bacterial communities at the (**A**) phylum level and (**B**) genus level, including (**C**) *Bacteroides*, (**D**) *Lachnospiraceae_NK4A136_group* and (**E**) *dgA-11_gut_group*. ^a,b^ Mean values with different letters are significantly different (*p* < 0.05).

The composition of the microbiota in cecal contents is shown in Figure 6. At the phylum level, Firmicutes and Bacteroidota were identified as the dominant phyla in the rabbit cecum (Figure 6A). Dietary supplementation with EEO and SI elevated the abundances of both Firmicutes and Bacteroidota compared with the Control group. The EEO and SI groups exhibited a marked increase in the relative abundance of *Bacteroides* relative to the Control group (Figure 6C; *p* < 0.05). It is noteworthy that the relative abundance of *Lachnospiraceae_NK4A136_group* was significantly increased in all treatment groups compared to the Control group (Figure 6D, *p* < 0.05), while the relative abundance of *dgA-11_gut_group* significantly decreased (Figure 6E, *p* < 0.05).

## 4. Discussion

EEO, an aromatic mixture derived from Eucalyptus spp., contains a mixture of flavonoids and triterpenoids [19]. Supplementation of drinking water with thyme, peppermint, and EEO enhanced growth performance in Ross 308 broiler chicks [20]. SI, as phytoestrogens, possess multiple health-promoting and disease-preventive activities [21]. Long-term exposure to SI enhanced growth performance in pigs [22,23]. The results of this study indicate that dietary supplementation with EEO and SI, either individually or in combination, can enhance body weight and ADG while reducing the F/G in rabbits. The combined supplementation group demonstrated the most significant improvement, achieving statistically superior outcomes compared to the Control group. Notably, during the 66–80 days phase, no significant changes were observed in ADG, ADFI, or F/G with EEO and SI supplementation, suggesting limited impact on production performance in the late fattening stage of meat rabbits. This beneficial effect on growth performance may be because EEO stimulates digestive gland secretions and enhances the activity of key digestive enzymes [24]. Daidzein downregulate the expression of the pro-inflammatory factors TNF-α and IL-1β in the intestines, preventing growth stasis caused by immune system stress [9]. Collectively, our findings demonstrate synergistic effects of EEO and SI co-supplementation in improving the growth performance of rabbits.

Carcass traits are generally correlated with dietary nutritional levels [25]. In the present study, the dressing percentage in the EEO group, SI group, and EEO + SI group was significantly higher than that in the control group, with the combined supplementation of EEO and SI exhibiting a synergistic effect on slaughter performance. This improvement may stem from EEO enhancing the rate of muscle deposition through dual mechanisms: (a) modulating growth axis hormones (GH/IGF-1) and inhibiting muscle degradation pathways (e.g., the ubiquitin-proteasome system); (b) activating the mTOR signaling pathway via bioactive compounds (1,8-cineole), thereby promoting muscle fiber hypertrophy [3]. Daidzein bind to hypothalamic estrogen receptors (ER-β), stimulating the release of growth hormones and enhancing protein deposition [9]. The elevated dressing percentage across treatment groups was related to improved ADG, as a higher ADG is typically associated with accelerated muscle protein synthesis and a reduced proportion of maintenance energy expenditure, ultimately increasing dressing yield. Supporting evidence showed that weaned dairy calves with ADG = 1.55 kg/d achieved 61.4% dressing percentage versus only 51.09% at ADG = 0.75 kg/d [26], consistent with our findings.

Consumer satisfaction with rabbit meat products is closely linked to quality attributes such as pH values, tenderness, and color. Meat quality largely depends on pH values, as acidity influences the capacity for glycogen storage in muscles and mitochondrial abundance within muscle fibers [27]. Shear force is negatively correlated with pH values, where lower values correspond to superior tenderness of the muscle [28]. Furthermore, elevated pH values inhibit myofibrillar contraction, consequently enhancing water-holding capacity [29]. In this study, the increase in pH_45min_ and the decrease in drip loss and shear force value across all treatment groups indicate that EEO and SI exert an improving effect on rabbit meat quality. Moreover, only the EEO + SI group exhibited a significant reduction in pH_45min_. Furthermore, the drip loss in the EEO + SI group was reduced by 24% and 18.36% compared to the EEO group and SI group, respectively. This demonstrates that combined dietary supplementation with EEO and SI yields superior effects on meat quality enhancement compared to individual supplementation. These findings are in accord with existing studies in the literature, confirming that a higher pH value is associated with better meat quality. Essential oils have been reported to inhibit lipid oxidation and improve meat quality [30]. Previous studies have shown that dietary supplementation with SI improves pH values and meat tenderness in broilers by reducing lipid peroxidation and enhancing antioxidant status [31], while also increasing intramuscular fat deposition (marbling) in fattening cattle [32]. The improvement in meat quality by EEO may be due to its anti-inflammatory and immune-enhancing properties that mitigate deterioration in quality induced by disease or stress. Meanwhile, the immunomodulatory and antioxidant effects of SI may also improve meat quality. Although this study confirmed that EEO and SI could improve rabbit meat quality, the underlying mechanisms linking their anti-inflammatory and immunomodulatory effects to quality enhancement warrant further investigation. Notably, supplementing EEO and SI to the diet increased the ether extract content of the rabbit meat, and this result was closely related to the improvement of the tenderness of the rabbit meat.

Serum biochemical parameters provide critical reference values for assessing metabolic status and health conditions. Serum ALT and AST levels are conventional biomarkers for evaluating hepatic injury [33]. LDH, a key glycolytic enzyme ubiquitous in cells, reflects enzyme leakage from cellular damage and necrosis; elevated levels may indicate cardiovascular disorders [34]. In addition, elevated triglyceride levels are correlated with cardiovascular diseases and constitute a pathological pathway for atherosclerotic conditions [35]. In this study, decreasing trends in ALT, AST, and LDH were observed across all treatment groups relative to the Control groups, although only the EEO + SI group exhibited a statistically significant reduction. Notably, dietary supplementation with EEO and SI significantly lowered serum triglyceride levels. Overall, the combined dietary supplementation of EEO and SI exhibits a synergistic effect on the improvement of serum biochemical parameters and provides protective effects on the liver and cardiovascular system of rabbits. The observed protection likely stems from the anti-inflammatory properties of EEO, coupled with SI’ mechanism of promoting reverse cholesterol transport to reduce the accumulation of cholesterol. However, investigations into such mechanisms remain relatively scarce; thus, further studies designed to validate these potential pathways are necessary.

Oxidative stress refers to a state in which the balance between oxidation and antioxidation in the body is disrupted. This imbalance can lead to metabolic disorders or partial functional impairments [36]. T-AOC maintains redox homeostasis by scavenging free radicals and preserving cellular membrane integrity [37]. GSH-Px and SOD constitute primary antioxidant defense components that protect cells against elevated levels of reactive oxygen species [38]. MDA, as an end-product of lipid peroxidation, reflects the extent of oxidative damage to cell membranes and serves as a key biomarker of oxidative stress [39]. In this study, dietary supplementation with EEO and SI increased serum T-AOC and GSH-Px levels, while decreasing MDA content in rabbits. The most pronounced effects were observed in the combined supplementation group. Furthermore, a synergistic effect on enhancing serum antioxidant capacity was demonstrated with the co-supplementation of EEO and SI in the diet. These improvements may have been due to the phenolic, flavonoid, and monoterpene compounds present in EEO that accelerate the clearance of reactive oxygen species by providing hydrogen atoms [40]. Flavonoiden activate transcription of catalase genes to promote the decomposition of hydrogen peroxide, thereby augmenting endogenous antioxidant enzyme systems [41]. Similarly, supplementing essential oils to the diet increased the T-AOC and SOD levels in broilers and reduced the MDA content [42]. Supplementing SI to the diet enhanced the antioxidant capacity of broilers and weaned piglets [22,31].

The small intestine serves as the primary site for nutrient digestion and absorption. Preservation of its structural integrity enhances the capacity for nutrient assimilation, thereby improving growth performance [43]. Intestinal morphology is a critical indicator for evaluating digestive-absorptive competence [44]. Increased villus height, coupled with reduced crypt depth, expands the intestinal absorptive surface area and facilitates efficient nutrient transport [45]. In this investigation, dietary supplementation with EEO, SI, or their combination enhanced small intestinal villus height and V/C ratio, with the combined supplementation group demonstrating more pronounced improvements. Furthermore, we observed a synergistic effect between the combined supplementation of EEO and SI on the enhancement of small intestinal villus height and V/C ratio. This structural enhancement correlates with functional benefits, as each 100 μm increase in villus height improves amino acid absorption by ~18% in rabbits [46]. These results suggest that one reason for the improvement in rabbit growth performance may be the enhanced morphology of intestinal tissues, which in turn improves nutrient absorption. Consistent with this hypothesis, supplementation with a blend of thyme, mint, and eucalyptus oils in broiler chicken diets reduced crypt depth while increasing the V/C ratio [20]. Moreover, dietary soy peptides ameliorated jejunal morphology in broilers [14]. The above findings are similar to the results of this study. The underlying mechanisms may involve the following aspects: (a) Essential oils activating TRPV ion channels to strengthen tight junctions, thereby preserving villus epithelial integrity [47]; (b) genistein (the bioactive aglycone of SI) inhibiting NF-κB signaling and suppressing iNOS and caspase-3 activities, thereby reducing enterocyte apoptosis [48].

The gut microbiota plays pivotal roles in nutrient digestion and absorption, exerting critical regulatory effects on host development [49]. This study confirmed that Firmicutes and Bacteroidota constituted the dominant phyla within the cecal microbiota, collectively accounting for >85% of total bacterial abundance across all experimental groups, consistent with previous findings [50,51]. *Bacteroides* competitively inhibits pathogen proliferation to maintain gut microbial homeostasis and prevent diarrhea [52]. Furthermore, *Lachnospiraceae_NK4A136_group* as a kind of butyrate- producing bacteria, could maintain the integrity of the intestinal barrier, inhibit inflammation, and prevent obesity [53]. At the genus level, the EEO and SI groups significantly increased the relative abundance of *Bacteroides* compared with the Control group. Meanwhile, the relative abundance of *Lachnospiraceae_NK4A136_ group* in each experimental group increased. This indicates an improvement in the intestinal health condition. In addition, the relative abundance of *dgA-11_gut_group* in each experimental group showed a significant decrease. In the goat experiment, it was found that the addition of a high proportion of straw significantly reduced the relative abundance of *dgA-11_gut_group*, accompanied by deterioration of intestinal health [54]. However, the specific function of this microbial component has not been directly validated through intervention experiments. Future research could prioritize the isolation and cultivation of strains, functional characterization of their metabolites, and exploration of their potential for therapeutic or nutritional applications in animal production. Complementary studies demonstrate that essential oils reduce pathogenic bacteria and promote probiotic growth through disrupting bacterial membrane integrity and inhibiting peptidoglycan synthesis [55]. Flavonoid compounds alleviate intestinal inflammation by suppressing lipopolysaccharide biosynthesis and pro-inflammatory cytokine production [56]. Thus, microbiota modulation may be linked to the anti-inflammatory properties of these supplements, although this causal relationship warrants further validation. Overall, dietary supplementation with EEO and SI synergistically improved intestinal health by enhancing small intestinal morphology and modulating gut microbiota.

## 5. Conclusions

Dietary supplementation with EEO and SI alone or in combination enhanced the growth performance of meat rabbits. This improvement was associated with increased serum antioxidant capacity, enhanced intestinal development, and alterations in serum biochemical parameters and gut microbiota composition. Furthermore, EEO and SI synergistically improved meat quality by elevating pH value, water-holding capacity, tenderness, and fat proportion. Additionally, the supplementation of EEO and SI increased the slaughter rate of rabbits, and showed a synergistic effect when used in combination. Overall, the combined supplementation of EEO and SI demonstrated the most effective outcomes. This study has two main limitations: (a) The entire trial lasted 35 days, which may not be sufficient to reflect long-term effects. (b) Only one concentration of EEO and SI was tested. Future research should focus on further refining the optimal concentration range to achieve better outcomes.

## Figures and Tables

**Table 1:** Basic diet composition and nutritional level (on an air-dried basis).

Ingredients (%)		Calculated Analysis of Nutrient Levels (%)	
Corn	16	Digestible energy (MJ/kg)	10.12
Wheat bran	18	Crude protein	16.85
Soybean meal	15	Crude fiber	15.42
Puffed soybean powder	2	Ether extract	6.2
Peanut hull powder	10	Ash	8.7
Groundnut stem meal	20	Calcium	1.12
Tea residue	13	Total phosphorus	0.72
Glutamic acid residue	2		
Premix ^a^	4		
Total	100		

^a^ Premix is provided per kilogram of diet: 10,000 IU vitamin A; 750 IU vitamin D3; 49 mg vitamin E; 1.7 mg vitamin K; 6.5 mg vitamin B1; 8.3 mg vitamin B2; 42 mg niacin; 1.1 mg folic acid; 22 mg pantothenic acid; 0.12 mg biotin; 75 mg iron; 15 mg copper; 55 mg zinc; 40 mg manganese; 0.15 mg cobalt; 0.3 mg iodine; 0.25 mg selenium.

**Table 2:** The effects of dietary supplementation with eucalyptus essential oil and soy isoflavones on the growth performance of meat rabbits at the end of the experiment (*n* = 30, 5 replicates in each group with 6 rabbits in each replicate).

Item	Control	EEO	SI	EEO + SI	*Q*-Value
BW (kg)					
45 days	1.16 ± 0.05	1.16 ± 0.03	1.16 ± 0.04	1.16 ± 0.03	/
65 days	1.88 ± 0.05 ^b^	1.95 ± 0.07 ^ab^	1.95 ± 0.04 ^ab^	2.02 ± 0.05 ^a^	2.04
80 days	2.47 ± 0.06 ^c^	2.60 ± 0.07 ^ab^	2.55 ± 0.05 ^bc^	2.64 ± 0.05 ^a^	1.34
ADG (g)					
45–65 days	37.34 ± 3.12 ^c^	41.32 ± 2.54 ^b^	41.58 ± 3.03 ^ab^	45.13 ± 1.83 ^a^	2.06
66–80 days	39.69 ± 2.53	43.46 ± 5.53	39.92 ± 2.75	41.02 ± 1.05	0.36
45–80 days	38.37 ± 1.17 ^c^	42.26 ± 1.87 ^ab^	40.85 ± 2.38 ^b^	43.32 ± 1.10 ^a^	1.33
ADFI (g)					
45–65 days	150.40 ± 11.54	147.89 ± 9.80	152.54 ± 6.65	156.40 ± 10.15	/
66–80 days	211.94 ± 20.54	226.65 ± 14.35	212.46 ± 19.62	211.01± 11.02	/
45–80 days	177.55 ± 6.44	182.63 ± 6.37	178.97 ± 11.58	180.49 ± 9.15	0.59
F/G (g/g)					
45–65 days	4.03 ± 0.07 ^a^	3.58 ± 0.13 ^b^	3.68 ± 0.15 ^b^	3.47 ± 0.25 ^b^	1.32
66–80 days	5.34 ± 0.27	5.25 ± 0.37	5.32 ± 0.21	5.15 ± 0.28	2.11
45–80 days	4.63 ± 0.15 ^a^	4.33 ± 0.16 ^b^	4.41 ± 0.11 ^ab^	4.21 ± 0.26 ^b^	1.44

Means within a row with different superscripts are significantly different (*p* < 0.05). *Q* > 1.15 represents a synergistic effect. When the *Q* value is negative, it is represented by “/”, and the same applies to the following tables and figures.

**Table 3:** Effects of dietary supplementation with eucalyptus essential oil and soy isoflavones on the slaughter performance of meat rabbits at the end of the experiment (*n* = 3 per group).

Item	Control	EEO	SI	EEO + SI	*Q*-Value
Live weight (kg)	2.44 ± 0.05	2.55 ± 0.10	2.56 ± 0.04	2.51 ± 0.10	0.68
Half-eviscerated weight (kg)	1.30 ± 0.04 ^b^	1.44 ± 0.02 ^a^	1.47 ± 0.05 ^a^	1.44 ± 0.03 ^a^	1.12
All eviscerated weight (kg)	1.19 ± 0.05 ^b^	1.34 ± 0.02 ^a^	1.36 ± 0.06 ^a^	1.33 ± 0.03 ^a^	1.06
Semi-clean slaughter rate (%)	53.11 ± 1.13 ^b^	56.74 ± 2.42 ^a^	57.42 ± 1.29 ^a^	57.38 ± 0.83 ^a^	1.25
Total eviscerated slaughter rate (%)	48.93 ± 1.20 ^b^	52.44 ± 2.17 ^a^	53.18 ± 1.50 ^a^	53.11 ± 0.80 ^a^	1.27

Means within a row with different superscripts are significantly different (*p* < 0.05). *Q* > 1.15 represents a synergistic effect.

**Table 4:** The effects of dietary supplementation with eucalyptus essential oil and soy isoflavones on the muscle quality traits and nutrient composition of meat rabbits at the end of the experiment (*n* = 3 per group).

Item	Control	EEO	SI	EEO + SI	*Q*-Value
Meat quality traits					
pH_45min_	6.58 ± 0.16 ^b^	6.75 ± 0.05 ^ab^	6.75 ± 0.26 ^ab^	6.89 ± 0.08 ^a^	1.85
pH_24h_	5.86 ± 0.06	5.84 ± 0.07	5.85 ± 0.05	5.89 ± 0.04	/
Cooking loss (%)	31.15 ± 3.63	29.76 ± 5.49	32.35 ± 3.27	27.10 ± 3.13	/
Drip loss (%)	4.18 ± 0.35 ^a^	2.75 ± 0.54 ^b^	2.56 ± 0.63 ^b^	2.09 ± 0.41 ^b^	1.72
Shear force value (N)	37.56 ± 2.59 ^a^	33.73 ± 2.17 ^ab^	32.29 ± 2.85 ^b^	30.54 ± 1.72 ^b^	1.96
Meat composition (%)					
Moisture	76.57 ± 0.69	77.88 ± 0.76	77.21 ± 1.13	77.64 ± 0.71	0.83
Crude protein	82.15 ± 1.90	83.63 ± 2.04	83.85 ± 1.30	83.61 ± 1.55	1.01
Ether extract	4.07 ± 0.67 ^b^	5.73 ± 0.45 ^a^	5.60 ± 0.63 ^a^	6.05 ± 0.56 ^a^	1.42
Ash	5.47 ± 1.01	5.58 ± 0.38	5.77 ± 0.65	5.32 ± 0.80	/

Means within a row with different superscripts are significantly different (*p* < 0.05). *Q* > 1.15 represents a synergistic effect.

**Table 5:** The effects of dietary supplementation with eucalyptus essential oil and soy isoflavones on the serum biochemical indices of meat rabbits at the end of the experiment (*n* = 3 per group).

Item	Control	EEO	SI	EEO + SI	*Q*-Value
TP (g/L)	57.03 ± 2.76	58.90 ± 2.23	60.43 ± 1.93	58.77 ± 2.96	0.99
ALB (g/L)	37.90 ± 1.56	38.70 ± 3.99	38.63 ± 1.80	40.13 ± 0.72	2.81
ALT (U/L)	63.17 ± 5.42 ^a^	57.17 ± 6.19 ^ab^	58.70 ± 7.53 ^ab^	50.47 ± 1.89 ^b^	2.18
AST (U/L)	56.83 ± 2.93 ^a^	54.48 ± 4.78 ^a^	51.75 ± 4.75 ^a^	42.70 ± 3.59 ^b^	6.10
LDH (U/L)	667.33 ± 41.63 ^a^	587.67 ± 42.74 ^ab^	553.33 ± 58.24 ^ab^	520.00 ± 99.92 ^b^	1.20
TC (mmol/L)	1.75 ± 0.13	1.68 ± 0.32	1.71 ± 0.37	1.81 ± 0.19	/
UREA (mmol/L)	4.29 ± 0.32	3.86 ± 0.10	3.90 ± 0.25	3.72 ± 0.54	1.41
TG (mmol/L)	0.68 ± 0.13 ^a^	0.28 ± 0.04 ^b^	0.30 ± 0.06 ^b^	0.30 ± 0.07 ^b^	1.18

Means within a row with different superscripts are significantly different (*p* < 0.05). *Q* > 1.15 represents a synergistic effect. TP: total protein; ALB: albumin; ALT: alanine transaminase; AST: aspartate aminotransferase; LDH: lactic dehydrogenase; TC: total cholesterol; UREA: urea nitrogen; TG: triglyceride.

**Table 6:** The effects of dietary supplementation with eucalyptus essential oil and soy isoflavones on the serum antioxidant capacity of meat rabbits at the end of the experiment (*n* = 3 per group).

Item	Control	EEO	SI	EEO + SI	*Q*-Value
T-AOC (U/L)	0.63 ± 0.02 ^b^	0.74 ± 0.02 ^a^	0.71 ± 0.03 ^a^	0.75 ± 0.04 ^a^	1.20
SOD (U/mL)	86.34 ± 5.03	95.90 ± 11.33	99.72 ± 6.09	96.26 ± 3.01	1.18
MDA (nmol/mL)	5.64 ± 0.22 ^a^	4.91 ± 0.41 ^b^	4.87 ± 0.38 ^bc^	4.27 ± 0.20 ^c^	2.00
GSH-Px (U/mL)	415.36 ± 16.33 ^b^	439.68 ± 18.52 ^ab^	437.12 ± 21.84 ^ab^	471.04 ± 13.62 ^a^	2.34

Means within a row with different superscripts are significantly different (*p* < 0.05). *Q* > 1.15 represents a synergistic effect. T-AOC: total antioxidant capacity; SOD: superoxide dismutase; MDA: malondialdehyde; GSH-Px: glutathione peroxidase.

## Data Availability

The original contributions presented in this study are included in the article. Further inquiries can be directed to the corresponding author.
